# Evaluation of tibial rotational axis in total knee arthroplasty using magnetic resonance imaging

**DOI:** 10.1038/s41598-020-70851-z

**Published:** 2020-08-21

**Authors:** Ji-Hoon Nam, Yong-Gon Koh, Paul Shinil Kim, Gihun Kim, Yoon Hae Kwak, Kyoung-Tak Kang

**Affiliations:** 1 Department of Mechanical Engineering, Yonsei University, 50 Yonsei-ro, Seodaemun-gu, Seoul, 03722 Republic of Korea; 2 Joint Reconstruction Center, Department of Orthopaedic Surgery, Yonsei Sarang Hospital, 10 Hyoryeong-ro, Seocho-gu, Seoul, 06698 Republic of Korea; 3 Department of Orthopaedic Surgery, the Bone Hospital, 67, Dongjak-daero, Dongjak-gu, Seoul, Republic of Korea; 4 Department of Orthopaedic Surgery, Severance Hospital, Yonsei University College of Medicine, Seoul, Republic of Korea

**Keywords:** Bone, Skeleton

## Abstract

Surgeon-dependent factors such as optimal implant alignment of the tibial component are thought to play a significant role in the outcome following primary total knee arthroplasty (TKA). In addition, tibial component malrotation is associated with pain, stiffness, and altered patellofemoral kinematics in TKA. However, measuring tibial component rotation after TKA is difficult. Therefore, the purpose of this study was to find a reliable method for positioning the tibial component in TKA. To investigate the morphology of the tibial plateau, 977 patients' knees (829 females and 148 males) were evaluated using MRI. The relationships between the femoral transepicondylar axis (TEA), Akagi line, posterior tibial margin (PTM), medial third of the tibial tubercle (MTT), and anatomical tibial axis (ATS) were investigated in this study. In addition, gender difference in tibial rotational alignment were evaluated. Relative to the TEA, the MTT and ATS were externally rotated by 0.5° ± 4.4° and 0.5° ± 5.4°, respectively, while Akagi line and PTM were internally rotated by 3.7° ± 4.5° and 9.9° ± 6.1°, respectively. Gender differences were found in MTT, Akagi line and ATS (P < 0.05). Our result showed that the rotational alignment led to notable variance between femoral and tibial components using fixed bone landmarks. The MTT and ATS axes showed the closest perpendicular aspect with projected TEA. And the MTT and Akagi axes showed the reduced variance. In addition, PTM is not a reliable landmark for rotation of the tibial component. Based on the results of this study, surgeons may choose the proper anteroposterior axis of the tibial component in order to reduce rotational mismatch and improve clinical outcomes.

## Introduction

In orthopedic field, total knee arthroplasty (TKA) is known as a cost-effective treatment which restore knee mobility important for quality of life^1^. However, this procedure can cause chronic pain and failure that may lead to revision surgery in some patients^1^. In addition, the overall function of TKA is dependent on factors such as soft tissue balancing, joint line restoration, and implant alignment^2^. Rotational malalignment may lead to tibiofemoral flexion–extension instability, patellar mal-tracking, patellofemoral pain, and premature wear in tibial inserts^3–5^. Previous studies have shown that the femorotibial malalignment of rotation bring higher rates of revision and improper clinical outcomes in patients^5,6^.

Many previous studies have established reliable and reproducible reference axes of internal–external rotation in femur condyles for more appropriate and accurate alignment^7^. The well-known reference axes include the transepicondylar axis (TEA), the posterior condylar axis, and the Whiteside line^7–9^. Among these axes, the TEA has been considered as a useful anatomic reference axis^7^. As a result, the TEA is mainly used in total knee surgery as the rotational reference when align the femoral component.

However, there have been considerable arguments regarding which rotational reference should be the standard for tibial alignment^10^. Traditionally, the anteroposterior (AP) axis is determined on tibial component during preoperative planning using various bony landmarks as references^11,12^. There are still arguments regarding whether the AP axis has the best reliability with the least variability, and also regarding the identification of the optimal AP axis of the tibial component^13^. Unfortunately, these references are different for each individual patient and thus are unreliable^14^. Therefore, most surgeons consider the footprint of the posterior cruciate ligament and anterior tuberosity of the tibia as reference points^6^. Three points of the tibial tubercle which were previously suggested for the rotation alignment of tibial component are the medial third, the medial border, and the most anteriorly prominent point of the tuberocity^5,10,14^. Nonetheless, these landmarks show great variance among patients^10,15^. And Varus knees with osteoarthritis (OA) always provide constrained flexion-extention mobility, and insufficient extension are observed in raw computed tomography (CT) data^16,17^. This could affect the alignment while surgery in femoral and tibial preparation^18^.

Virtual surgery has an advantage on the precise evaluation of AP alignment^19^. By aligning the mechanical axis of femur and tibia and by adjusting the internal–external rotation in the intended direction, malrotation of the femorotibial joint can be corrected^18^.

Therefore, in this study, we performed the evaluation of various reference AP axes on tibial component in the view of accuracy and variability using virtual surgery. The relationships between the femoral transepicondylar (TEA) axis, medial border of the tibial tubercle (Akagi line), posterior tibial margin (PTM), medial third of the tibial tubercle (MTT), and anatomical tibial axis (ATS) were investigated in this study. In addition, gender difference in tibial rotational alignment were evaluated.

We hypothesized that the line provided by the ATS methods is perpendicular to the TEA and shows low variability compared to TEA.

## Results

Differences in tibial rotation axis angle measured with TEA and the four different methods are summarized in Table 1. When compared to TEA, the MTT and ATS methods were externally rotated by 0.5° ± 4.4° and 0.5° ± 5.4°, respectively, whereas the Akagi line and PTM were internally rotated by 3.7° ± 4.5° and 9.9° ± 6.1°, respectively. The percentages of cases where each rotational axis differed by 3°, 5°, and 7° from the projected TEA are shown in Table 2. Compared to the TEA, MTT showed the lowest average values and percentage of outliers followed by ATS, Akagi, and PTM. The mean absolute deviations and standard deviation for the four rotational axes are summarized in Table 3. The MTT and Akagi methods provided the lowest values for both standard deviation and mean absolute deviation, followed by ATS, and PTM. Gender difference were found in the MTT, Akagi line and ATS methods (P < 0.05). However, there were no gender differences in PTM. Relative to the TEA, the tibial rotation axis angle was externally rotated by 0.8° ± 4.3° for females, and internally rotated by 1.1° ± 4.5° for males in MTT, whereas it was internally rotated by 3.4° ± 4.5° and 5.2° ± 4.6° for females and males, respectively, in Akagi line. In the ATS method, the tibial rotation axis angle was internally rotated by 1.2° ± 5.9° for females, and externally rotated by 0.8° ± 5.3° for males, whereas in PTM it was internally rotated in both cases (by 10.7° ± 6.6° for females and 9.8° ± 6.0° males) when compared to TEA.

**Table 1:** Comparison of anthropometric measurements between Korean males and females.

Parameter (angle)	Whole patients (n = 977)	Female (n = 829)	Male (n = 148)	P-value
	Mean ± SD (range)	Mean ± SD (range)	Mean ± SD (range)	
MTT and (⊥of TEA)	0.5 ± 4.4 (− 12.9, 13.8)	0.8 ± 4.3 (− 11.9, 13.8)	− 1.1 ± 4.5 (− 12.9, 12.3)	< 0.05
Akagi line and (⊥of TEA)	− 3.7 ± 4.5 (− 17.5, 9)	− 3.4 ± 4.5 (− 16.7, 9)	− 5.2 ± 4.6 (− 17.5, 8.6)	< 0.05
ATS and TEA	0.5 ± 5.4 (− 31.1, 33.2)	− 1.2 ± 5.9 (− 31.1, 24.5)	0.8 ± 5.3 (− 19.2, 23.2)	< 0.05
PTM and TEA	− 9.9 ± 6.1 (− 29.2, 20.9)	− 10.7 ± 6.6 (− 25.4, 20.9)	− 9.8 ± 6.0 (− 29.2, 18.2)	n.s.

*n.s.* non-significant.

**Table 2:** Percentage of outlier 3°–7° differed from projected TEA.

Parameter (angle)	3° (%)	5° (%)	7° (%)
MTT and (⊥of TEA)	48.5	24.7	12.0
Akagi line and (⊥of TEA)	63.4	42.8	25.3
ATS and TEA	55.4	31.5	17.2
PTM and TEA	92.1	83.8	72.3
	< 0.05	< 0.05	< 0.05

*n.s.* non-significant.

**Table 3:** Mean absolute deviation and standard deviation from projected TEA.

	Mean absolute deviation	Standard deviation
MTT and (⊥of TEA)	3.5	4.4
Akagi line and (⊥of TEA)	3.6	4.5
ATS and TEA	4.1^a^	5.4^a^
PTM and TEA	4.7^a^	6.1^a^

*n.s.* non-significant.

^a^Significant different from MTT.

## Discussion

The most important finding of this study is that there were various alternative lines for the tibia rotation axis measurement when using bony landmarks. It was hypothesized that the ATS method provides the most perpendicular and the least variable results compared to TEA. However, it was found that the MTT and ATS line was the most perpendicular line compared to the TEA line. And it was also found that MTT and Akagi line showed the least variance.

The relationship between the femoral and tibial components in rotational alignment is an important factor that influences overall function and durability in TKA^20,21^. On the femoral side, the TEA has been established as the mediolateral axis of the femur, and setting the femoral component parallel to the TEA is considered to be reasonable^7^. However, there is little consensus in previous studies regarding the ideal rotational alignment of the tibial component in TKA^22^. Significant variations in the final rotation can be observed depending on the techniques and landmarks^22^. In addition, those references are based on individual surgeons’ preferences and experiences, although various anatomic references have been recommended in order to determine the tibial orientation^7^. Therefore, it would be worth establishing a well-defined reference axis to determine tibial rotational orientation in TKA.

It was compared that how perpendicular to TEA the tibial rotational axis was for 977 Korean patients using 3D MRI. The TEA projection technique was applied on the tibia as described by Akagi et al.^7^ and Bonnin et al.^22^. The use of 3-D reconstructions in radiology for descriptive anatomical studies or examination of pathologic lesions is well guided in previous studies^7,22–24^. Using these techniques, the number of available subjects is larger, and their demographic data are accessible. The digitization of relevant points using a 3D rendering makes the measurement more accurate because the view of reconstructed knee has the ideal magnification and spatial orientation. Therefore, the advantages of these studies can be summarized as increasing the precision of measurements and the selection of suitable subjects^22^. Therefore, it was evaluated that the morphology of the proximal tibia using MRI and repeated all measurements in alignment with the four common reference axes: MTT, PTM, Akagi line, and ATS.

As previously mentioned, it was expected that the ATS method would provide the most perpendicular line with the least variance. Because, by using this method, landmarks of the knee was identified and the relationships within landmarks was demonstrated^25^. In this study, the most constant landmark was the geometrical center of the medial plateau^25^. The geometrical center of lateral plateau could also be derived from points on the cortex^25^. In the mediolateral dimension, it was equally constant but not in the anteroposterior dimension. The center of tibial tubercle was derived by for the same manner with small interobserver errors^25^. However, our results show that the ATS methods provided similar perpendicular lines compared to MTT, but with greater standard deviation. A previous study documented that the tibial component was externally rotated in 19° from femoral component when using tibial tubercle as a rotational reference. However, the number of specimens was seven and the study was formed on normal knees^15^. Akagi et al. measured the angle between different landmarks and a line perpendicular to the TEA on subjects. They found that an axis crossing from the medial border of the tubercle to the footprint of posterior cruciate ligament has the lowest variability^14^. However, non-osteoarthritic knees were used on this measurement and it is difficult to find the footprint of the posterior cruciate ligament on CT image of the patient with total knee arthroplasty. Furthermore, it could be difficult to identify the medial border of the ligamentum patellae after medial arthrotomy^10^. Previous studied have claimed that the tibial baseplate should be rotated externally to approximate the MTT in order to maximize function^2,21^. In fact, the MTT technique could lead to excessive external rotation of the tibial component^2,26^. Our results show that MTT was externally rotated by 0.5° ± 4.4°, a similar result to the one obtained by Wernecke, that showed 1.4° ± 4.9° externally rotated^12^. Some studies have suggested that the alignment of the tibial component with the center of PTM could be beneficial for patellofemoral tracking and anterior knee pain^22^. However, our results showed the greatest standard deviation and absolute deviation in PTM. Our results show that the standard deviation in PTM was ± 6.1°, while a previous study showed a similar result (± 5.1°)^22^.

This investigation document that using the axis from the MTT of the tibia as a rotational reference for the tibial alignment leads to better tibiofemoral alignment in extension than using different rotational axis. This representation is supported by the results of previous study where the trial component was rotated to a point 5° lateral to the tibial tubercle medial border^27^. The study of Uehara^26^ and Cobb’s^25^ also documented that anatomical axis was lateral of the medial border of the tibial tuberosity. Our study shows that MTT is still a reliable landmark that closely approximates the perpendicular axis of TEA. The MTT is slightly externally rotated. The patellofemoral tracking could be improved and other complications of tibial component related to internal rotation could be avoided by external rotation of tibia^28,29^. The complications caused by the malrotation of component have helped surgeons to decide what is clinically acceptable. Therefore, setting the tibial component to align with the MTT may be beneficial to the patellofemoral joint.

It was found that females had MTT axes that were more externally rotated than men for TEA. In addition, except for the PTM axis, gender differences were found in the tibial rotational axis for all methods. Although our results were limited to Korean patients, they suggest that MTT and ATS perpendicular aspect to TEA, and MTT and Akagi line low variances should be specially considered.

This study has some limitations. First, MRI scans were used to develop the 3D representations of the femur and tibia in this study, and there could be some errors in the computation model. Nevertheless, the reconstruction soft tissues such as the articular cartilage needs to be performed on MRI, and potential inaccuracies in 3D reconstructions could be reduced using protocols used in our previous studies^23,24^. Second, our population lacks ethnic diversity, and the results might differ in other populations. Third, supine MRI scans were performed, resulting in non-weight bearing, which does not engage the screw-home mechanism. This may result in small variations in flexion and rotation of the knee with alterations in tibiofemoral congruency. And the projected TEA axis varies with flexion and extension angle, multiple angle can be considered in further study. Fourth, there is imbalance in gender. But the population has imbalanced because female subjects are majority for TKA in Korea. The imbalance sample size could affect the assumption of equal variances and cause a general loss of power. For this reason, we use Welch’s test when the variance was unequal. The power was 99.8%. Finally, postoperative clinical outcomes were not considered in this study. Nevertheless, this study provides valuable information regarding which tibia component rotational axis corresponding TEA is the most perpendicular and shows the least variance.

In conclusion, this is the first study with a large sample that investigates the relationship between TEA and four widely used different tibia rotational axes using MRI. The tibial rotation axis from MTT of the tibial baseplate resulted in a reliable tibiofemoral alignment than the other rotational axes in full extension position. In addition, PTM is not a reliable landmark for rotation of the tibial component.

## Materials and methods

**Figure 1:**
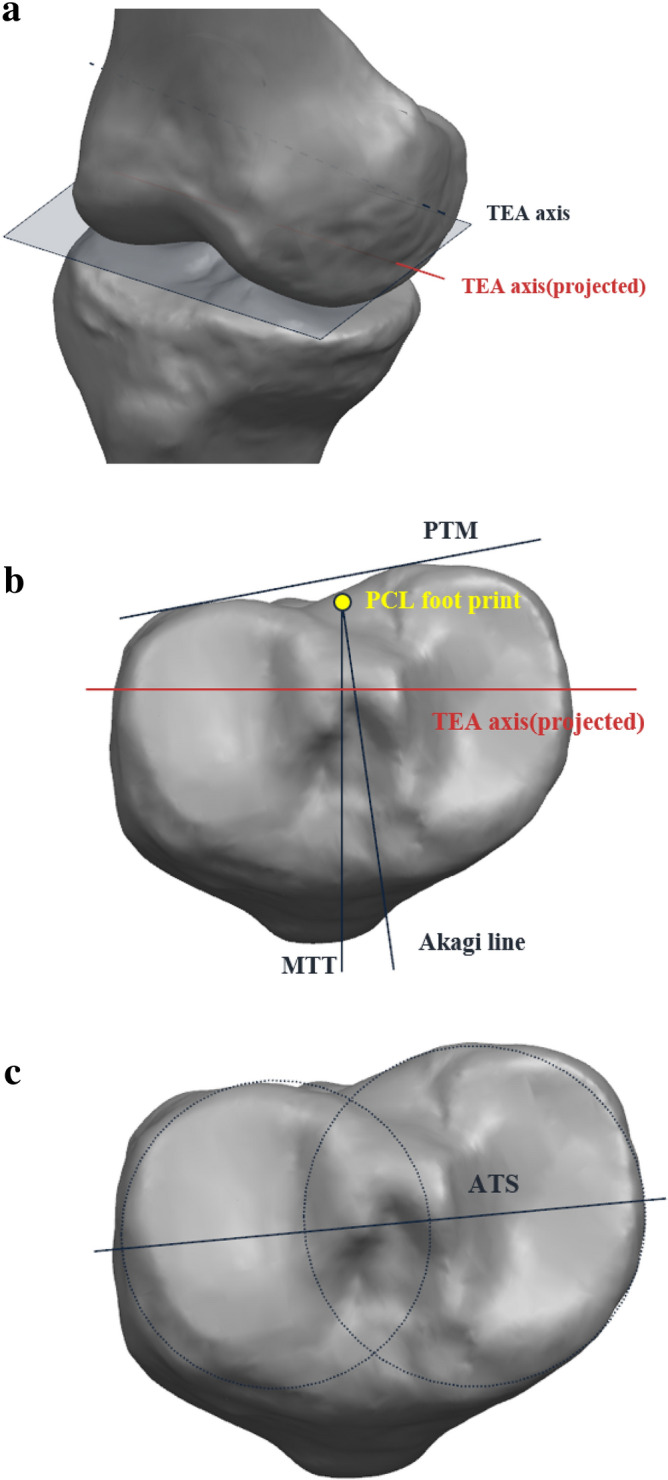
Schematic representation of (**a**) TEA, (**b**) PTM, MTT, Akagi line, and (**c**) ATS.

One thousand fifty-three MRI scans of patients undergoing TKA were investigated prior to primary TKA at our institution. Only patients with a history of osteoarthritis and TKA were included. All methods were performed in accordance with the relevant guidelines and regulations. The need for the written informed consent has waived by the ethics committee (Yonsei Sarang Hospital IRB). All patients had Kellgren and Lawrence grade 3 and 4. The Candidates with rheumatoid arthritis, severe bone defects in the proximal tibia, or a history of knee injuries or infections were excluded from this study. In the end, 977 patients (829 females and 148 males) were included in this study. The mean age of female and male patients was 69.5 ± 6.4 and 69.4 ± 6.7 years, respectively. The mean BMI for female and male patients was 30.2 ± 3.3 kg/m^2^ and 28.9 ± 3.1 kg/m^2^ (Table 4), respectively. There were no significant differences in demographics between male and female groups including age and BMI. The magnetic resonance imaging evaluation was approved by our institutional review board (IRB No. 18-DR-03). The approved protocol is measurement of knee joint anthropometric data using on 3-dimensional magnetic resonance imaging. The MRI scan using 1.5-T MRI scanner were performed (Achieva 1.5 T; Philips Healthcare, Best, Netherlands) with 1 mm slice thickness as high-resolution in the sagittal plane of the knee joint, while the hip and ankle joints scanning was performed in 5 mm slice thickness in the axial plane. The scanning was conducted using an axial proton-density sequence for the non-fat saturation condition. A high-resolution setting was used for the spectral pre-saturation inversion recovery sequence (echo time, 25.0 ms; repetition time, 3,590.8 ms; acquisition matrix, 512 × 512 pixels; number of excitations [NEX], 2.0; field of view, 140 × 140 mm). This MRI method used in patient-specific instruments allowed us to effectively obtain 3D reconstructed models. The MRI scans were imported into the software Mimics version 17.0 (Materialise, Leuven, Belgium) and segmented to develop 3D bony and cartilage models of the femur and tibia. A 3D reconstruction reproducibility analysis was performed using a method similar to previous studies^23,24^. On the femur, the TEA was defined using the lateral and medial condyles. The alignment of the three-dimensional MRI data was used for the femur-tibia alignment. The TEA was projected on the plane normal to the tibial mechanical axis (Fig. 1a). This plane was located on the proximal tibia. Then, four methods (PTM, MTT, Akagi, and ATS) commonly used as tibial rotation axis were defined (Fig. 1b,c). The PTM line was defined using the posterior condyles. This line contacted each condyle in the most posterior position^22^. The Akagi line was defined using the PCL foot print and medial border of the tibia tuberosity^7,14^. The PCL footprint was located on the middle of the posterior cruciate ligament attachment zone. The MTT axis was defined using the PCL footprint and medial third of the tibial tuberosity^10^. The axial slice containing the most anterior point of tuberosity was selected to define proximal–distal position of tuberosity. And the medial and lateral tuberosity points were picked using reconstructed 3D model and MRI. The one-third point was calculated as internally dividing point of medial and lateral tuberosity points. The PCL footprint was shared by the Akagi and MTT lines. Accordingly, as shown in Fig. 1b, the MTT line was rotated more externally than the Akagi line. The ATS method was defined using the two centers of the medial and lateral tibial plateaus, located on the center of a circle fitted to each plateau^25^. The angle between these four lines and the projected TEA was measured^18^. All measurements were performed by a well-trained observer. To assess the intra- and inter-observer variability, 3D MRI scans of 50 female and 50 male patients were re-assessed more than a week after the first measurement by the same observer and a second observer. The inter-observer errors of MTT, Akagi, ATS, PTM with respect to TEA were 0.82, 0.83, 0.92, and 0.92, respectively, and the intra-observer errors of MTT, Akagi, ATS, PTM with respect to TEA were 0.95, 0.95, 0.87, and 0.93, respectively.

**Table 4:** Comparison of the age, BMI and varus-valgus deformity between Korean males and females.

Parameter	Whole patients (n = 977)	Female (n = 829)	Male (n = 148)	p-value
	Mean ± SD (range)	Mean ± SD (range)	Mean ± SD (range)	
Age	68.9 ± 6.4 (50, 95)	69.4 ± 6.7 (52, 89)	69.5 ± 6.4 (50, 95)	n.s.
BMI (kg/m^2^)	30.0 ± 3.3	30.2 ± 3.3	28.9 ± 3.1	n.s.
Varus deformity	8.6 ± 5.3 (− 15.7, 25.6)	8.7 ± 5.4 (− 15.7, 25.6)	7.9 ± 5.0 (− 10.1, 19.8)	n.s.

*n.s.* non-significant.

### Statistical analysis

A post hoc power analysis was performed for TEA and MTT angles between the two groups using the G power 3.1 software. The statistical power was calculated with the alpha value 0.05. The power calculated was 99.8%. After the power analysis, the test of significance was conducted on SPSS (version 18.0; SPSS, Chicago, IL, USA). The *t-*test was used to determine the significance differences between genders in 829 female and 148 male patients. The chi-squared test was used to compare the percentages of cases where each rotational axis differed by 3°, 5°, and 10° from the projected TEA. The one-way ANOVA was used to compare mean absolute deviation and variance. Bonferroni method was used as post hoc analysis. The significant level was 0.05 (P < 0.05).
